# Structural equation modeling to explore putative causal factors for chronic fatigue in childhood cancer survivors: a DCCSS LATER study

**DOI:** 10.1007/s11764-024-01738-5

**Published:** 2025-02-28

**Authors:** Adriaan Penson, Ioan Gabriel Bucur, Iris Walraven, Martha A. Grootenhuis, Heleen Maurice-Stam, Margriet van der Heiden-van der Loo, Helena J. H. van der Pal, Andrica C. H. de Vries, Dorine Bresters, Marry M. van den Heuvel-Eibrink, Sebastian Neggers, Birgitta A. B. Versluys, Marloes Louwerens, Saskia M. F. Pluijm, Nicole M. A. Blijlevens, Eline van Dulmen-den Broeder, Leontien C. M. Kremer, Tom Heskes, Jacqueline Loonen, Hans Knoop

**Affiliations:** 1 Center of Expertise for Cancer Survivorship, Department of Hematology, Radboud University Medical Center, Nijmegen, The Netherlands; 2 Department of Data Science, Institute for Computing and Information Sciences, Radboud University Nijmegen, Nijmegen, The Netherlands; 3 Department for Health Evidence, Radboud University Medical Center, Nijmegen, The Netherlands; 4 Princess Máxima Center for Pediatric Oncology, Utrecht, The Netherlands; 5 Department of Pediatric Oncology, Erasmus Medical Center, Rotterdam, The Netherlands; 6 Wilhelmina Children’s Hospital, UMCU, Utrecht, The Netherlands; 7 Department of Medicine, Erasmus Medical Center, Section Endocrinology, Rotterdam, The Netherlands; 8 Department of Internal Medicine, Leiden University Medical Center, Leiden, The Netherlands; 9 Department of Pediatric Oncology/Hematology, Amsterdam University Medical Center, Amsterdam, The Netherlands; 10 Department Pediatric Oncology, Emma Children’s Hospital, University of Amsterdam, Amsterdam, The Netherlands; 11 Department of Medical Psychology, Amsterdam University Medical Centers, University of Amsterdam, Amsterdam Public Health Research Institute, Amsterdam, The Netherlands; 12 Cancer Center Amsterdam, Amsterdam, The Netherlands

**Keywords:** Cancer survivors, Causality, Childhood cancer, Etiology fatigue, Survivorship

## Abstract

**Purpose:**

To explore putative causal relations for chronic fatigue (CF) in childhood cancer survivors (CCS) using structural equation modeling (SEM).

**Methods:**

Interrelationships between factors that were previously associated with CF and their causal relation with CF were investigated using SEM and causal discovery methodology. A bootstrap method was used to ascertain how robust each finding was, presenting the percentage of times that each discovered edge was found in 1000 bootstrap samples as a measure of confidence (with > 50% needed to be confident in a found edge).

**Results:**

1927 CCS (51.7% male) with a mean age of 35 years (SD 9) participated in the study (23.6% reported CF). Results indicated that sex had a putative causal effect on CF (bootstrap confidence 81%), while CF was causally linked to helplessness, physical activity, pain, BMI, and sleep problems (bootstrap confidence 82%, 78%, 78%, 51%, and 51%, respectively). The relation between CF and depression was found to be two-way (bootstrap confidence 68%), indicating a reciprocal relation or the presence of a latent confounder. The same applied to the relations between CF and anxiety, self-esteem, and social functioning, but this could not be confirmed with high confidence (bootstrap confidence < 50%).

**Conclusion:**

This study provides insight into the complex etiology of CF and could give guidance in the development of appropriate prevention and/or intervention strategies for CF in CCS.

**Implications for Cancer Survivors:**

Results show the consequences of having CF and could help to understand the impact it has on daily life.

**Supplementary Information:**

The online version contains supplementary material available at 10.1007/s11764-024-01738-5.

## Background

One in four long-term childhood cancer survivors (CCS) suffer from chronic fatigue (CF) [1]. CF is a debilitating symptom that affects quality of life [2]. Previous studies have shown multiple variables to be related to CF in CCS, for example, childhood cancer diagnosis (e.g., brain tumors), treatment-related variables (e.g., cranial irradiation), demographics (e.g., female sex), and various lifestyle and psychological variables (e.g., physical activity level or depression) [3–5].

In a recent publication, we determined the relationship of CF with aforementioned variables in one model and showed that lifestyle and psychosocial factors were most strongly associated with CF in CCS [6]. However, using multiple regression analysis, we only determined associations between CF and the other variables. To better understand the relation between CF and these other factors in CCS, a next step would be to indicate how these factors might possibly be causally related. To adequately investigate causal relationships between factors, longitudinal prospective studies are the golden standard. However, such studies are often costly and time-consuming and were lacking in CCS until now.

Alternatively, in the current study, we employed innovative data-driven causal discovery methods to ascertain putative causal relationships using readily available cross-sectional data [6]. Doing so, we aimed to explore putative causality between previous determined associated factors. While we cannot assess all potentially causal factors in a single model, we aim to provide some structure in the large web of factors associated with CF in CCS and to gain more insight in the possible underlying causal relations.

A variety of data-driven computational methods for causal discovery have been developed in the past decades, aiming to find underlying causal relations from (cross-sectional) observational data [7, 8]. These methods start from the fundamental assumption that there is an underlying structural causal (equation) model describing the generic causal mechanism that generated the observed variables. The key notion behind causal discovery is that these (very general) causal models have statistical implications (even) in cross-sectional data that can be tested and falsified, and we can therefore exclude any causal models that do not explain the data. Even if the exact causal model cannot usually be recovered, these methods can still be used to discover or suggest plausible causal links among the variables interest, often from a combination of observational data and background knowledge [9].

Structural equation modeling (SEM) is a multivariate analysis technique that can be used to examine potentially causal relationships between factors using cross-sectional data [10–12]. Here, we focus on the *structural* part of an SEM approach, with which we want to characterize the causal relationships among the observed variables. Structural causal models represent a very general way of describing the causal relations between variables in a system that can incorporate hidden confounding, cycles, and arbitrary functional relationships. We used the SEM approach to investigate hypothesized relations based on background knowledge and expert opinion and to analyze possible causal relations for CF in CCS using the causal structure output of constraint-based causal discovery (BCCD).

The BCCD algorithm, proposed by Claassen and Heskes [13], is a Bayesian approach for robust constraint-based causal discovery in which Bayesian scoring is used to determine the most likely causal (sub)structures that could have generated the data, while conflict resolution is used to arrive at a single, most reliable output model. In other words, various possible causal pathways are tested, but only the most likely ones, based on the observed data probability distribution, are retained. In addition to assuming an underlying structural causal model, BCCD relies on causal faithfulness, which means that any constraints found in the data (in the form of conditional independencies) correspond to constraints on the causal structure. Finally, parametric assumptions about the data distribution (e.g., multivariate Gaussian) are necessary in order to define a likelihood and Bayesian score over the different causal models. More details regarding the BCCD algorithm are given in supplementary Text Box 1.

The aim of the current study was to explore putative causal relations between CF and factors that were previously shown to be associated with CF, which might lead to new insights regarding possible consequences (and causes) of CF in CCS.

## Methods

### Study design and participants

This cross-sectional study, which was part of the Dutch Childhood Cancer Survivor Study on Late Effects (DCCSS LATER) part 2 [14], extends on previously published methodology and results [6]. In short, study participants were included from the DCCSS LATER cohort, a Dutch nationwide cohort including 5-year cancer survivors [15]. Participants, who were still alive and living in the Netherlands at time of data collection (2017–2020) and who were not lost to follow-up or had previously declined to participate in any research, were invited to participate in the study (*N* = 4735). Participants for the current study were 18 years of age or older and gave written informed consent to participate. The DCCSS LATER fatigue study was approved by the Medical Research Ethics Committee of the Amsterdam UMC (registered at toetsingonline.nl, NL34983.018.10).

### Data collection

Data were collected during a LATER outpatient clinic visit, which took place between 2017 and 2020 in one of the seven pediatric oncology centers in the Netherlands. If preferred, participants could complete questionnaires at home (on paper or digitally). Fatigue severity was assessed with the *fatigue severity subscale* of the Checklist Individual Strength (CIS) [16]. The CIS has satisfying psychometric properties in CCS [17]. CF was defined as reporting severe fatigue (a score of 35 or higher on the *CIS fatigue severity* subscale [18]) with a self-reported duration of at least 6 months. The duration was assessed in a separate item next to the CIS.

In a previous study, we found that out of a large pool of possible CF-related variables, the following lifestyle and psychosocial factors to be associated with CF: sex, BMI, physical activity, anxiety, depression, pain, self-esteem, feelings of helplessness, social functioning, and sleep problems [6]. We focused on these factors in the current study. In the period 2017–2020, data were collected during a clinic visit (or by digital questionnaires when a visit was not possible) as follows:

- To calculate body mass index (BMI), height and weight were measured manually during the clinic visit (or self-reported when a clinic visit was not possible).
- The European Prospective Investigation into Cancer and Nutrition (EPIC) physical activity questionnaire [19] was used to assess weekly physical activities. EPIC items were used to categorize participants using the four-point physical activity index as proposed by Wareham et al. [19] into being (A) physically active, (B) moderately physically active, (C) moderately physically inactive, or (D) physically inactive.
- The hospital anxiety and depression scale (HADS) [20, 21] was used to assess feelings of anxiety and depression. Participants were indicated as having (sub)clinical anxiety or depression based on HADS scale scores (subscale score ≥ 8).
- Pain was measured on a 6-point Likert scale, ranging from having no pain (score of 1) to very much pain (score of 6).
- The Rosenberg self-esteem scale (RSES) [22, 23] was used to assess self-esteem. Items were added up (total score ranging 10–40), with a higher score reflecting higher self-esteem.
- The illness cognition questionnaire (ICQ) [24, 25] helplessness subscale was used as an indication of feelings of helplessness. Six items of the subscale were added up (range 6–24), with higher scores reflecting more feelings of helplessness, related to the childhood cancer.
- The TNO (Netherlands Organization for Applied Scientific Research) and AZL (Leiden University Medical Centre) Questionnaire for Adult’s Quality of Life (TAAQOL) social functioning domain [26] was used as an indication of the participant’s social functioning. Subscale scores were transformed to a 0–100 scale following instructions described elsewhere [26], with higher scores reflecting better social functioning.
- The Pittsburg sleep quality index (PSQI) [27, 28] was used to assess sleep quality. A global score that reflects overall sleep quality was computed following instructions described elsewhere [27, 28].

Diagnosis and treatment data of primary diagnoses and all recurrences of the CCS participants were collected from medical records by data managers using a uniform protocol [29]. More details regarding data collection can be found elsewhere [14, 30].

### Statistical analyses

Descriptive statistics were calculated using the diagnosis and treatment related variables to describe the group of participants. Chi-square analyses were performed to analyze differences between participants and non-participants, with Cramér’s *V* effect sizes used to indicate little (≤ 0.1), low (> 0.1), medium (> 0.3), and high (> 0.5) differences between groups.

A combination of both expert opinions (of authors AP, IW, HK, and JL) and the BCCD algorithm [13] was used to determine the potential causal relations between CF and its associated lifestyle and psychosocial factors. An SEM analysis was performed to evaluate novel causal and two-way relations suggested by BCCD (Box 1). More specifically, the following steps were conducted:

1. Using available literature [31–34] and expert opinions of the authors, we have outlined possible causal and two-way relationships between CF and all factors. All pathways between the study variables were indicated as being (A) causal, (B) two-way, or (C) not (directly) related. All hypothesized causal and two-way relations are presented in Supplementary Table 1 and describe the initial SEM (Model 1).
2. We ran the BCCD algorithm [13] on the data in two contexts:
3. Incorporating information from the initial SEM from step 1. This was done to limit the number of putative causal relations to be tested, ensuring the algorithm to focus on important discrepancies between the data and the expert model. Any pathway hypothesized as “not related” was enforced missing when running BCCD. Pathways hypothesized as causal were given as input to BCCD as background knowledge. Note that the pathways could be overruled and turned into two-way relations whenever BCCD found inconsistencies with the data. Along the same lines, BCCD could turn hypothesized two-way relations in the initial SEM into causal relations. This analysis resulted in a new, partially data-driven, model (Model 2).
4. Incorporating minimal background knowledge: Sex precedes all other variables and CF precedes all other variables (except sex) as its definition indicates that participants have had fatigue symptoms for at least 6 months where for the other variables participants were asked to indicate symptoms over the past few weeks. However, as other symptoms might have been present for a longer time period as well, we assume that two-way relationships with CF are possible, and could even be due to a third variable (latent confounder) that precedes both. BCCD was not given any additional information, meaning the algorithm tested most causal pathways against the data. This analysis resulted in a final, mainly data-driven, model (Model 3).
5. To ascertain how robust each finding was, we used the bootstrap method [35] to resample our data 1000 times and reran step 2 on each bootstrap sample. We reported the percentage of times that each relation was found in the bootstrap samples as a confidence measure, with > 50% as a majority decision threshold assumed to indicate adequate confidence in the relation. Overall model fit, evaluated using the *Bayesian information criterion* (BIC) score [36] and the and the *root mean square error of approximation* (RMSEA) [37], was used to indicate whether the model improved compared to the expert SEM from step 1, Both measures take into account both goodness-of-fit and model complexity (lower BIC score indicates improvement, as does lower RMSEA).

Details regarding assessment and categorization of variables included in the SEM analysis can be found in Supplementary Table 2. The regression estimates reported in Supplementary Table 4 represent risk differences, which are accompanied by standard error estimates based on the expected information matrix [38]. Missing data (no pattern observed) were imputed using multiple imputation (Markov chain Monte Carlo method, 20 imputed datasets) [39–41]. The correlation coefficients in the imputed datasets were combined using Rubin’s rules [42] to get a pooled correlation matrix that was used as input to the BCCD algorithm. *R* [38] *(lavaan and RUcausal packages* [43, 44]) was used for the statistical analyses.

**Box 1** Definitions of causal and two-way relations used in the current study

**Table:** 

Causal relation: Factor A is a plausible cause for factor B
Two-way relation: Factor A and factor B are dependent; one or both of the following could be true.
- Factor A and factor B have a cyclical relationship (A causes B and B causes A);
- A latent confounder is present between factor A and factor B

## Results

Participant characteristics (*n* = 1927) are presented in Table 1. Leukemia was the most prevalent childhood cancer diagnosis (35.3%) and 87.5% of the participants had been treated with chemotherapy (with or without additional radiotherapy). Mean age of the participants was 35 (SD = 9) years and 51.7% were male. Almost one-fourth of the participants were identified with CF (23.6%). A flowchart with the participants inclusion process and a comparison with non-participants is shown in Supplementary Fig. 1 and Supplementary Table 3, respectively. Participants were more often female (48.3% vs. 39.9%), had more often received a combination of chemotherapy and radiotherapy (33.2% vs. 24.4%), and had more often received a hematopoietic stem cell transplantation during childhood cancer (6.8% vs. 3.9%) compared to non-participants.

**Table 1:** Characteristics of participants

Characteristic	Participants (*n* = 1927)	
	*n*	%
Sex		
Male	996	51.7
Female	931	48.3
Age at assessment (years)		
Mean (SD)	35.1 (9.3)	
18–29	599	31.1
30–39	737	38.2
≥ 40	591	30.7
CF		
Yes	454	23.6
No	1473	76.4
Age at diagnosis (years)		
Mean (SD)	6.7 (4.7)	
0–4	886	46.0
5–9	519	26.9
10–14	414	21.5
15–17	108	5.6
Primary childhood cancer diagnosis ^a^		
Leukemia	678	35.3
Non-Hodgkin lymphoma ^b^	234	12.1
Hodgkin lymphoma	135	7.0
CNS	177	9.2
Neuroblastoma	111	5.8
Retinoblastoma	10	0.5
Renal tumors	220	11.4
Hepatic tumors	17	0.9
Bone tumors	109	5.7
Soft tissue tumors	141	7.3
Germ cell tumors	65	3.4
Other and unspecified ^c^	30	1.6
Period of childhood cancer diagnosis		
1963–1969	29	1.5
1970–1979	255	13.2
1980–1989	607	31.5
> 1990	1036	53.6
Childhood cancer treatment ^d^		
Surgery only	131	6.8
Chemotherapy, no radiotherapy	1047	54.3
Radiotherapy, no chemotherapy	100	5.2
Radiotherapy and chemotherapy	640	33.2
No treatment/treatment unknown	9	0.5
Hematopoietic Stem cell transplantation		
Yes	131	6.8
No	1783	92.5
Unknown	13	0.7
Cancer recurrence		
No	1675	86.9
Yes	252	13.1
Educational level		
Low	240	13.3
Middle	774	42.8
High	795	43.9
Missing	118	-
Employment status		
Employed	1538	85.1
Not employed	269	14.9
Missing	120	-
Relationship status		
In a relationship	1266	78.6
Not in a relationship	344	21.4
Missing	317	-
BMI		
Healthy weight	993	53.2
Underweight	54	2.9
Overweight	588	31.5
Obesity	230	12.3
Missing	62	-
Physical activity index		
Inactive	93	5.3
Moderately inactive	402	23.1
Moderately active	413	23.7
Active	836	47.9
Missing	183	-
(Sub)clinical anxiety		
No	1304	80.3
Yes	320	19.7
Missing	303	-
(Sub)clinical depression		
No	1492	92.0
Yes	130	8.0
Missing	305	-
Poor sleeper		
No	1176	64.2
Yes	655	35.8
Missing	96	-
Pain total score (range 1–6)		
Mean (SD)	2.0 (1.2)	
Missing	44	
Self-esteem total score (range 10–40)		
Mean (SD)	32,8 (5.6)	
Missing	294	
Helplessness total score (range 6–24)		
Mean (SD)	7.8 (3.1)	
Missing	326	
Social functioning total score (range 0–100)		
Mean (SD)	87.2 (18.4)	
Missing	248	

*BMI* body mass index, *CF* chronic fatigue, *SD* standard deviation

^a^Diagnostic groups included all malignancies covered by the third edition of the International Classification of Childhood Cancer (ICCC-3) as well as multifocal Langerhans cell histiocytosis

^b^Includes all morphology codes specified in the ICCC-3 under lymphomas and reticuloendothelial neoplasms, except for Hodgkin lymphomas. Also includes multifocal Langerhans cell histiocytosis

^c^Includes all morphology codes specified in the ICC-3 under other malignant epithelial neoplasms and malignant melanomas and other and unspecified malignant neoplasms

^d^Treatment data included primary treatment and all recurrences

BIC and RMSEA scores of the three analyzed models (the hypothesized model, the partially data-driven model and the mainly data-driven model) indicated decisive strength of evidence in favor of the mainly data-driven model. A comparison of the goodness-of-fit for the three models is shown in Table 2. All three models are “close” to the data in terms of RMSEA [45], but adjusting the initial model (Model 1) decreased the error by 30% for Model 2 and 35% for Model 3. Moreover, the BIC score of the BCCD-adjusted models also improved significantly. We obtained a Bayes factor (*K*) of 1.2 × 10^5^ decisive strength of evidence in favor of Model 3 (the final model) relative to Model 1 (the initial model), suggesting the data are (approximately) a hundred thousand times more likely under Model 3 rather than under Model 1 [37].

**Fig. 1:**
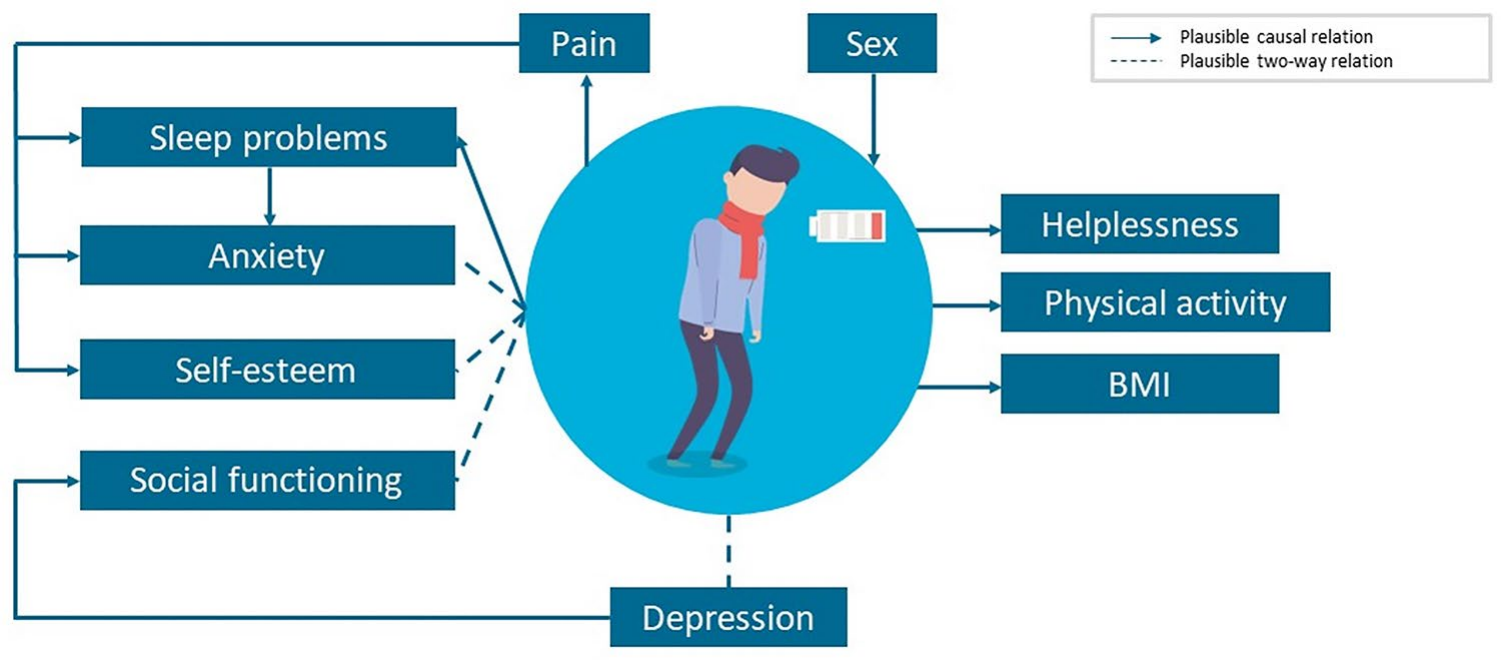
Path diagram showing (causal) relations between chronic fatigue and associated factors after incorporating BCCD output. Diagram shows plausible causal and two-way relations of Chronic Fatigue. Directed edge indicates plausible causal relationship (confirmed or adjusted by BCCD with > 50% confidence), dotted edge indicates a potential two-way relation between variables (the two-way relation of CF and depression was confirmed by BCCD with >50% confidence, the other two-way relations were hypothesized by the experts and not confirmed or adjusted by BCCD with >50% confidence). Note: Diagram is a simplified version of the found relations in the final SEM, not all relations between associated factors are shown (see Supplementary Table 4 for more details about all found relations)

We also report the SEM output of Model 1 (the hypothesized model) and Model 3 (the final model) in Supplementary Table 4, including bootstrap confidence percentages of all analyzed relations. Edge orientations after incorporating the BCCD output are shown in Table 3 (focusing on CF) and Supplementary Table 5 (all possible relations). Sex was found to be putative causal factor of CF, with a bootstrap confidence of 81%, while CF was found to be a putative causal factor for more feelings of helplessness (with a bootstrap confidence of 82%), lower physical activity index (78% bootstrap confidence), more pain (78%), a higher BMI (51%), and more sleeping problems (51%). The relation between CF and depression was found to be two-way (with a bootstrap confidence of 68%). The results also suggest a possible two-way relation between CF and anxiety, self-esteem, and social functioning, but these could not be confirmed with high confidence (all relations <50% bootstrap confidence). A graph showing all relations in the final BCCD adjusted model (Model 3) is shown in Supplementary Figure 2. A simplified graphical version of the results is presented in Fig. 1, where a path diagram shows just the interrelationships between CF and the studied associated factors.

**Table 2:** Comparison of model fit for the analyzed structural equation models

Model fit measure	Model 1 (expert-driven)	Model 2 (partially data-driven)	Model 3 (mainly data-driven)
RMSEA	0.05472	0.03848	0.03552
BIC score	88,442.86	88,422.43	88,419.48

*RMSEA* root mean square error of approximation (lower is better), *BIC* Bayesian information criterion (lower is better)

Both the RMSEA and BIC are used to assess how well a model fits the data, while also penalizing models that are too complex. The conventional cutoff values of RMSEA (.01, .05, .08 and .10) distinguish between excellent, close, fair, mediocre, and poor models, respectively [45]. A difference bigger than 10 for the BIC indicates “decisive” or “conclusive” evidence in favor of the model with smaller BIC [36, 37]

**Table 3:** Hypothesized causal and two-way relations for CF and associated factors and adjusted relations after BCCD data-driven output

Hypothesized relations (model 1)	Relations BCCD data-driven output (model 3)
CF ← sex	CF ← sex
CF ↔ pain	CF → pain
CF ↔ BMI	CF → BMI
CF ↔ physical activity	CF → physical activity
CF ↔ sleep problems	CF → sleep problems
CF ↔ social functioning	CF ↔ social functioning
CF ↔ self-esteem	CF ↔ self-esteem
CF ↔ depression	CF ↔ depression
CF ↔ anxiety	CF ↔ anxiety
CF ↔ helplessness	CF → helplessness

*BCCD* Bayesian constraint-based causal discovery, *BMI* body mass index, *CF* chronic fatigue

Hypothesized relations are based on expert opinions of authors AP, IW, HK, and JL in combination with available literature [31–34]. CF precedes all other variables (except sex) as its definition indicates that participants have had fatigue symptoms for at least 6 months and for the other variables participants were asked to indicate symptoms over the past few weeks. However, as other symptoms might have been present for a longer time period as well, we assume that two-way relationships with CF are possible, and could even be due to a third variable (latent confounder) that precedes both

Direction of arrow shows direction of (hypothesized) causality:

A → B = hypothesized causal pathway from A to B

A ← B = hypothesized causal pathway from B to A

A ↔ B = hypothesized two-way relation

## Discussion

In the current study, we explored plausible causal relations for CF in CCS using an innovative statistical approach, with the aim to gain more insight into putative causes and consequences of CF in CCS. These insights could be helpful to determine which factors are most likely affected by CF and would thus require extra attention during clinic visits when CF is present.

### Clinical implications

Sex was found to be a causal factor for CF. Although sex will not actively cause symptoms of fatigue, we believe sex to act as an indirect causal factor. The precise nature of this causal relation remains a question, but women tend to more often experience fatigue compared to men, which might be related hormonal/biological differences between the sexes [46, 47]. Furthermore, sex differences have been associated with (risk factors for) other outcomes as well, e.g., neurological and cardiovascular [48, 49]. This emphasizes the need to provide care that is tailored to the person’s situation and needs, which might be different for men and women. Alternatively, social and cultural differences might play a role here, for example women with CF might be more prone to participating in a study compared to men with CF, or women might be less affected by a certain stigma that comes with fatigue.

CF was found to be a causal factor for reduced physical activity. Concordantly, in patients with osteoarthritis fatigue was shown to be a strong predictor for reduced physical activity, while among the elderly, fatigue was suggested to be the cause for reduced physical activity [50, 51]. The causality seems plausible as people who experience severe fatigue symptoms might have little energy or less motivation to actively engage in sports activities or perform energy consuming daily activities. On the other hand, previous studies showed that interventions aimed at increasing physical activity also decreased fatigue symptoms [52]. This suggests that, although CF might cause one to be less physically active as was found in the current study, a reversed causal relation might also be plausible, such that increasing physical activity might decrease CF symptoms. In addition, CF was found to be related to an increase in BMI which is possibly related to the relation between CF and physical activity, as physical activity and BMI are also related. In the current study, we assumed CF to precede all other variables (except sex), since it is the only variable exhibiting chronicity. Therefore, the possibility of any other variable (except sex) causing CF was not considered.

Our results also show that a causal effect of CF on pain is plausible. In the current study, 32.4% of the participants with CF reported to have rather much (24.7%) or (very) serious pain (7.7%), compared to 8.7% in the non-CF participants. This means that almost one-third of the CCS with CF experience some form of pain. However, details regarding the pain’s nature, i.e., acute or chronic pain, or the duration of the pain symptoms remains unknown, which makes the interpretation of the exact relation between pain and CF difficult. Whether a reduction of fatigue symptoms also leads to a clinically relevant decrease in the level of pain symptoms remains to be determined, e.g., by conducting an intervention study aimed at fatigue management in CCS and assessing its effect on pain. Studies in other patient populations have suggested a reduction in pain to possibly reduce symptoms of fatigue, which might indicate the relation between CF and pain to be the other way around [53, 54]. Future studies might explore this causal pathway in CCS in more detail.

As a note regarding all found two-way relations, we simply do not know the precise pathway between the factors. Factors could reinforce each other, or a latent confounder could be in play, but with the current analyses we did not find strong evidence for the presence of a causal pathway. Nevertheless, regardless of the precise relationships between these symptoms, it is known they are related to CF in CCS [6]. More studies are needed to confirm or explore these pathways further.

### Study limitations

The following needs to be taken into account when interpreting the results. Although SEM and the BCCD algorithm are adequate techniques for analyzing causal structure in a large network of variables and for investigating possible causalities using cross-sectional data, they rely on multiple model assumptions such as linearity and Gaussianity. Also, we assumed CF to precede all other variables (except sex) as its definition indicates that participants have had fatigue symptoms for at least 6 months where for the other variables participants were asked to indicate symptoms over the past few weeks. Still, other symptoms might have been present for a longer time period, and possibly preceding CF, as well. Any findings should be confirmed in prospective studies, wherever possible, using longitudinal data.

Another limitation regards the differences between participants and non-participants, indicating the possibility of selection bias. However, effect sizes were small, making us believe these differences to not affect the generalizability of the results (Cramér’s *V* effect sizes ≤ 0.13).

Finally, we chose > 50% for the bootstrap confidence percentages as the threshold for incorporating the reliable BCCD output into the expert SEM. We believe this majority decision threshold to reflect a simple choice that adequately indicates confidence in a newly discovered relation. Had we chosen a stricter threshold (for example 75%), we would have incorporated fewer data-driven relations into the SEM, thereby missing out on several relations that further improved the model fit.

## Conclusion

The current study presents plausible causal and two-way relations for CF in CCS. The results give more insight into the complexity of CF and its possible causes and consequences in CCS, which could offer some guidance for future studies in the development of prevention or intervention strategies.

## Supplementary Information

Below is the link to the electronic supplementary material.

Supplementary file1 (DOCX 254 KB)

## Data Availability

The data that were analyzed in this article were provided by the DCCSS-LATER consortium under license. Data will be shared on request to the corresponding author with permission of the DCCSS-LATER consortium.
