# Supplementary material for: Structural equation modeling to explore putative causal factors for chronic fatigue in childhood cancer survivors: a DCCSS LATER study
Source: J Cancer Surviv. 2025 Feb 28;20(4):1367–76. doi: 10.1007/s11764-024-01738-5 (PMC13375848; doi:10.1007/s11764-024-01738-5)
Supplement: Supplementary file 1 — Supplementary file1 (DOCX 254 KB) [file 11764_2024_1738_MOESM1_ESM.docx]

Supplementary material.

**Supplementary Text Box 1. BCCD (a Bayesian approach to causal inference) [1].**

Causal discovery refers to the task of inferring causal structure from data, or from a combination of data and expert knowledge. While inferring causality is typically done by employing longitudinal studies, there is an extensive body of work in the artificial intelligence field that has proved how causality can be inferred even from cross-sectional data [2]. The general idea for much of this methodology is to recognize certain correlation (conditional dependence) patterns in the data that can only be produced by certain causal models. The most classic example is the so-called *v*-structure, called that way because it corresponds to a structure that looks like a ‘v’, where two causes have a common effect.

Consider the following example, as previously presented by Bucur in [3], in which we want to understand the causal mechanism behind a wet lawn in a typical household yard. We know that the wet lawn is related to the on/off state of a sprinkler and to whether it rained that day, since we observed that both of these events are associated with a wet lawn. At the same time, we found no relationship between the state of the sprinkler and the presence of rain, since the sprinkler is only turned on during the weekend, without any regard for weather conditions. The final piece of the puzzle that leads to the *v*-structure conclusion is that, if the lawn is wet, and we know that the sprinkler is on, it becomes less likely that it is raining. Conversely, if the lawn is wet and it is raining, it becomes less likely that the sprinkler is on, as the presence of rain 'explains away’ any effect of the sprinkler, i.e., the lawn is already wet because of the rain. This phenomenon in which two independent causes (‘Sprinkler’ and ‘Rain’) show a correlation when conditioning upon the value of their common effect (‘Wet Lawn’) is called the *explaining away effect*. The pattern of having two independent variables that become dependent when conditioning on a third variable that is associated to both can only be produced by the *v*-structure in *Example 1* (d).


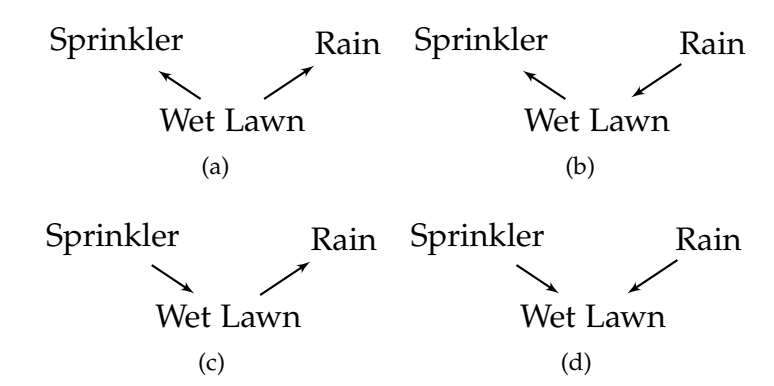


*Example 1.* From *[3].* Out of these four possible causal mechanisms, only the one (d) can produce the correlation pattern corresponding to the ‘explaining-away’ effect.

The BCCD algorithm is used to infer causal relationships from observational data in the presence of latent confounders. Key assumptions involve the existence of an underlying structural causal model describing, in very general terms, how the variables in our system are related to each other causally. In addition to assuming an underlying structural model, BCCD relies on causal faithfulness, which means that any constraints found in the data (in the form of conditional independencies) correspond to constraints on the causal structure. Finally, we make the parametric assumption that the data (approximately) follows a multivariate Gaussian distribution, which enables us to make use of the Bayesian Gaussian equivalent (BGe) score for scoring the various causal models [4]. In the lines that follow, we present a high-level description of how the BCCD algorithm works.

**The BCCD algorithm**

In: Database D over variables V, background information I

Out: causal relationships, causal graph

1. Search Stage (for finding the edges in the graph)

- Start from a fully connected graph over variables V.
- For each subset of at most five nodes, perform Bayesian scoring on all possible (causal) substructures using the BGe score. Given each substructure, record a list of their structural implications (S) and a list of their causal implications (C), noting how reliable these statements are given the BGe scores.
- Add the structural implications from the background information to S and sort the list in descending order by reliability.
- For each pair of (remaining) adjacent nodes, check in S if the reliability score for removing the edge, corresponding to causal structure in which the edge does not exist between the pair of nodes, is higher than a specified threshold. Repeat the process until all pairs of nodes have been processed.
- Return the skeleton of the graph, where each remaining (association) edge means that the corresponding pair of variables cannot be separated, i.e., they are conditionally dependent for all (explored) conditioning sets.

1. Inference Stage (for orienting the edges in the graph)

- Start from the skeleton obtained in the first stage.
- Add the causal implications from the background information to C and sort the list in descending order by reliability.
- For each causal implication in C above a specified confidence threshold, orient the skeleton graph accordingly.
- Return the list of most reliable adjacencies and causal relationships, together with the accompanying causal graph.
- Note that the causal graph will almost always include parts that cannot be fully identified from the data and background knowledge, meaning that in the end we will obtain an equivalence class of causal models that are indistinguishable and can explain the given information just as well.

**Supplementary Figure 1. Flowcharts of CCS and sibling study participants**


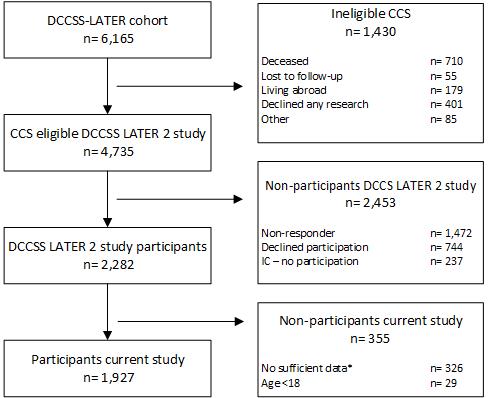


**Sufficient data to determine fatigue status: at least 7 of the 8 CIS fatigue severity items completed (with one missing value, the mean of the remaining completed items was imputed) + duration of fatigue symptoms completed (if fatigue severity subscale score ≥35).*

**Supplementary Table 1. Hypothesized causal and two-way relations for CF and associated factors.**

| Study variable | Hypothesized causal relations | Hypothesized two-way relations |
| --- | --- | --- |
| CF | CF ← sex | CF *↔* BMI  CF *↔* pain  CF *↔* physical activity  CF *↔* sleep problems  CF *↔* social functioning  CF *↔* self-esteem  CF *↔* depression  CF *↔* anxiety  CF *↔* helplessness |
| Sex | Sex *→* CF  Sex *→* sleep problems  Sex *→* self-esteem  Sex *→* depression  Sex *→* anxiety  Sex *→* helplessness  Sex *→* pain  Sex *→* BMI | n/a |
| BMI | BMI ← sex  BMI *→* self-esteem | BMI *↔* CF  BMI *↔* physical activity  BMI *↔* sleep problems  BMI *↔* depression |
| Anxiety | Anxiety ← pain  Anxiety ← sex  Anxiety *→* social functioning  Anxiety *→* sleep problems | Anxiety *↔* CF  Anxiety *↔* helplessness  Anxiety *↔* self-esteem  Anxiety *↔* depression |
| Depression | Depression ← sex | Depression *↔* CF  Depression *↔* anxiety  Depression *↔* helplessness  Depression *↔* pain  Depression *↔* physical activity  Depression *↔* sleep problems  Depression *↔* social functioning  Depression *↔* self-esteem  Depression *↔* BMI |
| Pain | Pain *→* sleep problems  Pain *→* social functioning  Pain *→* self-esteem  Pain *→* anxiety  Pain *→* helplessness  Pain ← sex | Pain *↔* CF  Pain *↔* physical activity  Pain *↔* depression |
| Physical activity | Physical activity *→* self-esteem  Physical activity *→* helplessness  Physical activity ← social functioning  Physical activity ← sleep problems | Physical activity *↔* CF  Physical activity *↔* depression  Physical activity *↔* pain  Physical activity *↔* BMI |
| Sleep problems | Sleep problems *→* physical activity  Sleep problems *→* social functioning  Sleep problems ← sex  Sleep problems ← self-esteem  Sleep problems ← anxiety  Sleep problems ← pain | Sleep problems *↔* CF  Sleep problems *↔* depression  Sleep problems *↔* helplessness  Sleep problems *↔* BMI |
| Self-esteem | Self-esteem *→* sleep problems  Self-esteem *→* social functioning  Self-esteem ← pain  Self-esteem ← BMI  Self-esteem ← physical activity  Self-esteem ← sex | Self-esteem *↔* CF  Self-esteem *↔* helplessness  Self-esteem *↔* depression  Self-esteem *↔* anxiety |
| Social functioning | Social functioning *→* physical activity  Social functioning ← sleep problems  Social functioning ← self-esteem  Social functioning ← anxiety  Social functioning ← pain | Social functioning *↔* CF  Social functioning *↔* helplessness  Social functioning *↔* depression |
| Helplessness | Helplessness ← pain  Helplessness ← physical activity  Helplessness ← sex | Helplessness *↔* CF  Helplessness *↔* self-esteem  Helplessness *↔* depression  Helplessness *↔* social functioning  Helplessness *↔* anxiety  Helplessness *↔* sleep problems |

*Abbreviations: CF=Chronic Fatigue, BMI=Body Mass Index.*

*Hypothesized relations are based on expert opinions of authors AP, IW, HK and JL in combination with available literature [5-8]. Direction of arrow shows direction of hypothesized causality:*

*A → B = hypothesized causal pathway from A to B*

*A* ← *B = hypothesized causal pathway from B to A*

*A ↔ B = hypothesized two-way relation*

*If no edge was present between parameters, no (unmediated) relation was hypothesized.*

**Supplementary Table 2. Overview of data collection, -categories and -availability of variables included in SEM analysis of CCS participants (n=1927).**

| **Variable of interest** | **Type of questionnaire or questionnaire item (when applicable)** | **Categories** | **Data availability (% missing)** |
| --- | --- | --- | --- |
| BMI | Height (m) and weight (kg) measured during clinic visit. BMI was calculated using the formula: weight (in kg) / (height (in m))^2^ | Underweight: BMI <18.5  Healthy weight: BMI between 18.5 and 24.99  Overweight: BMI between 25 and 29.99  Obesity: BMI ≥30 | n=1865 (3%) |
| Pain | How much pain did you experience in the past four weeks? Answer options: none (1), very mild, mild, some, much, very much (6). | Total pain score (range 1-6) | n=1883 (2%) |
| Physical activity | EPIC physical activity questionnaire items were used to categorize participants using the four-point physical activity index as proposed by Wareham et al. [9]. | *Inactive*: sedentary job and no recreational activity  *Moderately inactive*: sedentary job with <0.5h recreational activity per day or standing job with no recreational activity  *Moderately active*: sedentary job with 0.5-1h recreational activity per day or physical job with no recreational activity  *Active*: sedentary job with >1h recreational activity per day or standing job with >0.5h recreational activity per day or physical job with at least some recreational activity or heavy manual job.  Recreational activity was defined as activities of MET (Metabolic Equivalent) scores ≥4, as described in the compendium of physical activities (this includes biking and doing sports activities) [10]. | n=1744 (9%) |
| Anxiety | The outcomes of the seven items of the HADS anxiety subscale were added up and the total score was used to indicate person’s as having anxiety yes/no. | No anxiety: HADS anxiety subscale score <8  Anxiety: HADS anxiety subscale score ≥8 | n=1624 (16%) |
| Depression | The outcomes of the seven items of the HADS depression subscale were added up and the total score was used to indicate person’s as having depression yes/no. | No depression: HADS depression subscale score <8  Depression: HADS depression subscale score ≥8 | n=1622 (16%) |
| Social functioning | TAAQOL social functioning domain items were added up and linearly transformed to a 0-100 scale following instructions described elsewhere [11] (higher scores reflecting better social functioning) | TAAQOL social functioning domain score (continuous) | n=1679 (13%) |
| Self-esteem | Ten items of the RSES were added up (total score ranging 10-40), with a higher score reflecting higher self-esteem | RSES total score (continuous) | n=1633 (15%) |
| Helplessness | Subscale of the ICQ (helplessness) was calculated by adding up the six items of the subscale (score ranging 6-24). Higher score indicates more helplessness. | ICQ subscale helplessness (continuous) | n=1601 (17%) |
| Sleep quality | Seven PSQI component scores and total scores were calculated using scoring instructions described elsewhere Buysse et al. [12]. To compute the global score, the 7 component scores were added up. If at least 5 of the 7 component scores were present, a mean of the non-missing components was used to impute the missing value(s), as proposed by Beck et al. [13] | Good sleeper: PSQI total score ≤5  Poor sleeper: PSQI total score >5 | n=1831 (5%) |

*Abbreviations: BMI=Body Mass Index; EPIC=European Prospective Investigation into Cancer and Nutrition; HADS=**Hospital Anxiety and Depression Scale; TAAQOL=* *TNO (Netherlands Organization for Applied Scientific Research) and AZL (Leiden University Medical Centre) Questionnaire for Adult’s Quality of Life; RSES= Rosenberg Self-Esteem Scale; ICQ= Illness Cognition Questionnaire; PSQI=**Pittsburg Sleep Quality Index*. *The ordered categorical variables, as well as the binary variable CF, are treated as continuous in the statistical analyses.*

**Supplementary Table 3. Comparison CCS participants vs. non-participants.**

| **Characteristic** | **Participants**  **(n=1927)** | **Non participants (n=2064) *** | **P-value ^e^** | **ES ^f^** |
| --- | --- | --- | --- | --- |
|  | **N (%)** | **N (%)** |  |  |
| Sex  Male  Female | 996 (51.7)  931 (48.3) | 1241 (60.1)  823 (39.9) | <0.001 | 0.09 |
| Year of birth  <1960  1960 – 1969  1970 – 1979  1980 – 1989  ≥1990 | 21 (1.1)  152 (7.9)  502 (26.1)  740 (38.4)  512 (26.5) | 21 (1.0)  151 (7.3)  516 (25.0)  789 (38.2)  587 (28.5) | 0.11 | 0.05 |
| Age at diagnosis (years)  0-5  5-10  10-15  15-18 | 886 (46.0)  519 (26.9)  414 (21.5)  108 (5.6) | 977 (47.3)  564 (27.3)  393 (19.1)  130 (6.3) | 0.24 | 0.03 |
| Primary childhood cancer diagnosis ^a^  Leukemia  Non-Hodgkin lymphoma ^b^  Hodgkin lymphoma  CNS  Neuroblastoma  Retinoblastoma  Renal tumors  Hepatic tumors  Bone tumors  Soft tissue tumors  Germ cell tumors  Other and unspecified ^c^ | 678 (35.2)  234 (12.1)  135 (7.0)  177 (9.2)  111 (5.8)  10 (0.5)  220 (11.4)  17 (0.9)  109 (5.7)  141 (7.3)  65 (3.4)  30 (1.6) | 684 (33.1)  237 (11.5)  148 (7.2)  244 (11.8)  108 (5.2)  14 (0.7)  224 (10.9)  25 (1.2)  112 (5.4)  152 (7.4)  86 (4.2)  30 (1.5) | 0.35 | 0.06 |
| Period of childhood cancer diagnosis  1963-1969  1970-1979  1980-1989  >1990 | 29 (1.5)  255 (13.2)  607 (31.5)  1036 (53.8) | 18 (0.9)  255 (12.4)  631 (30.6)  1160 (53.1) | 0.15 | 0.04 |
| Childhood cancer treatment ^d^  Surgery only  Chemotherapy, no radiotherapy  Radiotherapy, no chemotherapy  Radiotherapy and chemotherapy  No treatment/treatment unknown | 131 (6.8)  1047 (54.3)  100 (5.2)  640 (33.2)  9 (0.5) | 244 (11.8)  1163 (56.3)  125 (6.1)  503 (24.4)  23 (1.1) | <0.001 | 0.13 |
| Hematopoietic stem cell transplantation  Yes  No  Missing | 131 (6.8)  1783 (92.5)  13 (0.7) | 81 (3.9)  1974 (95.6)  9 (0.5) | 0.001 | 0.07 |
| Cancer recurrence  No  Yes | 1675 (86.9)  252 (13.1) | 1821 (88.2)  243 (11.8) |  | 0.02 |

**Non-participants were invited to participate but did not return or complete the fatigue questionnaire (non-responders or missing fatigue questionnaire data). In flowchart in Supplementary Figure 1, non-participants DCCSS LATER 2 (n=2.453) and non-participants current study (n=355) are added up, minus 744 CCS who declined participation and who were therefore not analyzed.*

*^a^ Diagnostic groups included all malignancies covered by the third edition of the International Classification of Childhood Cancer (ICCC-3) as well as multifocal Langerhans cell histiocytosis.*

*^b^ Includes all morphology codes specified in the ICCC-3 under lymphomas and reticuloendothelial neoplasms, except for Hodgkin lymphomas. Also includes multifocal Langerhans cell histiocytosis.*

*^c^ Includes all morphology codes specified in the ICC-3 under other malignant epithelial neoplasms and malignant melanomas and other and unspecified malignant neoplasms.*

*^d^ Treatment data included primary treatment and all recurrences.*

*^e^ Chi-Square test*

*^f^ Cramér’s V effect size (<0.1=little, 0.1=low, 0.3=medium, 0.5=high).*

**Supplementary Table 4. Output SEM analyses.**

|  | **Initial SEM ^a^** | | | **Final SEM ^b^** | | | |
| --- | --- | --- | --- | --- | --- | --- | --- |
| **RMSEA** | **0.05472** | | | **0.03552** | | | |
| **BIC** | **88442.86** | | | **88419.48** | | | |
| **Causal relations (regressions)** | **Estimate** | **SE** | **p-value** | **Estimate** | **SE** | **p-value** | **Confidence in edge** |
| Chronic fatigue ~  Female sex | 0.107 | 0.018 | <0.001 | 0.146 | 0.019 | <0.001 | 80.8% |
| Anxiety ~  Female sex  Sleep problems  Pain  Self-esteem | 0.565  -  0.865  - | 0.158  -  0.066  - | <0.001  -  <0.001  - | 0.049  0.238  0.209  -0.306 | 0.130  0.023  0.059  0.013 | 0.709  <0.001  <0.001  <0.001 | <50%  <50%  <50%  74.1% |
| Depression ~  Female sex | 0.201 | 0.133 | 0.132 | 0.108 | 0.137 | 0.428 | <50% |
| Sleep problems ~  Chronic fatigue  Female sex  Pain  Anxiety  Self-esteem | -  0.313  0.571  0.251  -0.091 | -  0.126  0.054  0.021  0.014 | -  0.013  <0.001  <0.001  <0.001 | 1.507  0.231  0.558  -  -0.143 | 0.166  0.128  0.056  -  0.012 | <0.001  0.070  <0.001  -  <0.001 | 50.7%  <50%  -  <50% |
| Pain ~  Chronic fatigue  Female sex | -  0.400 | -  0.054 | -  <0.001 | 0.950  0.266 | 0.061  0.051 | <0.001  <0.001 | 77.7%  89.4% |
| Self-esteem ~  Female sex  Pain  Physical activity  BMI | -1.384  -1.068  0.138  -0.018 | 0.254  0.103  0.111  0.021 | <0.001  <0.001  0.217  0.406 | -1.589  -0.604  0.041  0.038 | 0.244  0.104  0.129  0.025 | <0.001  <0.001  0.749  0.134 | 83.2%  <50%  <50%  <50% |
| Social functioning ~  Pain  Anxiety  Self-esteem  Sleep problems  Depression | -0.571  -0.859  1.057  -0.545  - | 0.323  0.128  0.080  0.132  - | 0.078  <0.001  <0.001  <0.001  - | -0.508  -0.040  -  -0.109  -3.089 | 0.325  0.143  -  0.132  0.158 | 0.118  0.781  -  0.408  <0.001 | <50%  <50%  -  <50%  61.4% |
| Physical activity ~  Sleep problems  Social functioning  Chronic fatigue | 0.003  0.003  - | 0.008  0.001  - | 0.680  0.011  - | 0.013  0.001  -0.402 | 0.008  0.001  0.059 | 0.103  0.658  <0.001 | <50%  <50%  78.4% |
| Helplessness ~  Female sex  Pain  Physical activity  Depression  Chronic fatigue | 0.170  0.737  -0.280  -  - | 0.130  0.056  0.063  -  - | 0.191  <0.001  <0.001  -  - | 0.115  -  -0.189  0.398  1.436 | 0.117  -  0.061  0.022  0.160 | 0.327  -  0.002  <0.001  <0.001 | <50%  -  <50%  56.3%  82.2% |
| BMI ~  Female sex  Chronic fatigue | 0.331  - | 0.206  - | 0.108  - | 0.171  1.215 | 0.208  0.245 | 0.410  <0.001 | <50%  51.1% |
| **Two-way relations (covariances)** |  |  |  |  |  |  |  |
| Chronic fatigue ~~  Anxiety  Depression  Sleep problems  Self-esteem  Social functioning  Physical activity  Helplessness  BMI  Pain | 0.424  0.487  0.161  -0.627  -0.942  -0.046  0.354  0.162  0.161 | 0.032  0.028  0.023  0.049  0.131  0.008  0.027  0.037  0.012 | <0.001  <0.001  <0.001  <0.001  <0.001  <0.001  <0.001  <0.001  <0.001 | 0.165  0.596  -  -0.721  -0.708  -  -  - | 0.027  0.032  -  0.057  0.138  -  -  - | <0.001  <0.001  -  <0.001  <0.001  -  -  - | <50%  68.1%  -  <50%  <50%  -  -  - |
| Anxiety ~~  Depression  Self-esteem  Helplessness | 5.601  -9.699  3.191 | 0.258  0.467  0.234 | <0.001  <0.001  <0.001 | 2.222  -  -0.224 | 0.160  -  0.153 | <0.001  -  0.143 | 64.7%  -<50% |
| Depression ~~  Sleep problems  Pain  Self-esteem  Social functioning  Physical activity  Helplessness  BMI | 0.931  0.888  -8.935  -9.815  -0.247  3.709  0.628 | 0.136  0.083  0.404  0.802  0.054  0.207  0.228 | <0.001  <0.001  <0.001  <0.001  <0.001  <0.001  0.006 | 1.134  0.345  -9.457  -  -0.138  -  0.166 | 0.141  0.067  0.427  -  0.063  -  0.262 | <0.001  <0.001  <0.001  -  0.028  -  0.527 | <50%  <50%  55.3%  -  <50%  -<50% |
| Sleep problems ~~  Helplessness  BMI | 0.535  0.215 | 0.158  0.277 | 0.001  0.438 | 0.015  -0.201 | 0.153  0.280 | 0.920  0.473 | <50%  <50% |
| Pain ~~  Physical activity  Helplessness | -0.099  - | 0.028  - | <0.001  - | -0.061  0.446 | 0.025  0.065 | 0.014  <0.001 | <50%  57.0% |
| Self-esteem ~~  Helplessness  Social functioning | -6.423  - | 0.376  - | <0.001  **-** | -2.226  13.351 | 0.255  1.727 | <0.001  <0.001 | <50%  55.9% |
| Social functioning ~~  Helplessness | -9.281 | 0.941 | <0.001 | -7.723 | 0.899 | <0.001 | <50% |
| Physical activity ~~  BMI | -0.352 | 0.099 | <0.001 | -0.300 | 0.097 | 0.002 | <50% |

*CF=Chronic Fatigue, BMI=Body Mass Index. Significant edge: p<0.05. Hyphens (-) denote that a type of relation is missing in one of the SEMs (because the other type of relation was present).*

*^a^ Hypothesized relations based on literature and expert opinion of authors AP, IW, HK and JL (Model 1)*

*^b^ We ran BCCD causal discovery algorithm using minimal background knowledge (Model 3) and adjusted the initial model accordingly. A bootstrap resampling method was conducted to determine confidence levels of all edges in the model (percentage of times an edge was discovered in the 1000 bootstrap samples) and to determine possible new edges based on these confidence levels (new edges with a confidence of >50% were included in the final model).*

**Supplementary Table 5. Changes made to the hypothesized model after incorporating BCCD output.**

| **Edge orientation in hypothesized model** | **Edge orientation after incorporating BCCD output** | **Bootstrap confidence in edge** |
| --- | --- | --- |
| CF ↔ physical activity  CF ↔ BMI  CF ↔ helplessness  CF ↔ sleep problems  CF ↔ pain  Depression ↔ helplessness  Depression ↔ social functioning  Anxiety → sleep problems  Pain → helplessness  Self-esteem → social functioning | CF → physical activity  CF → BMI  CF → helplessness  CF → sleep problems  CF → pain  Depression → helplessness  Depression → social functioning  Sleep problems → anxiety  Pain ↔ helplessness  Self-esteem ↔ social functioning | 78.4 %  51.1 %  82.2 %  50.7%  77.7 %  56.3 %  61.4 %  51.4 %  57.0 %  55.9 % |

*CF=Chronic Fatigue. After incorporating the BCCD output, the RMSEA improved significantly from 0.5472 to 0.3552 (lower is better). Moreover, the BIC score improved significantly from 88442.86 to 88419.48 (lower is better), corresponding to a Bayes factor of approximately 1.2 × 10^5^. The table shows the edge orientations that were changed after BCCD output incorporation. Direction of arrow shows direction of (hypothesized) causality:*

*→ = causal relation*

*↔ = two-way relation*

**Supplementary Figure 2. Graphics showing relations of the final, BCCD adjusted model (Model 3).**

1. **Figure showing all causal relations**

*
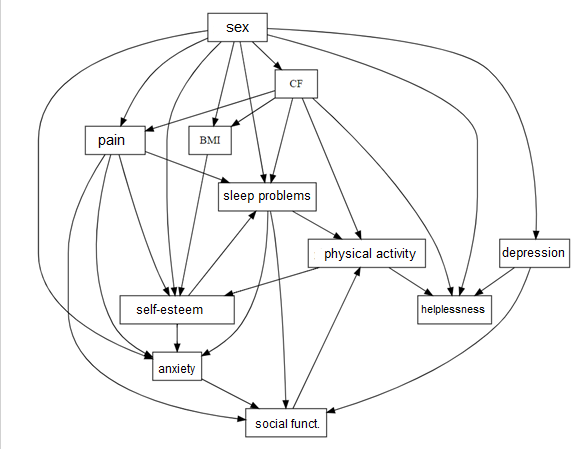
*

1. **Figure showing all causal and two-way relations.**

*
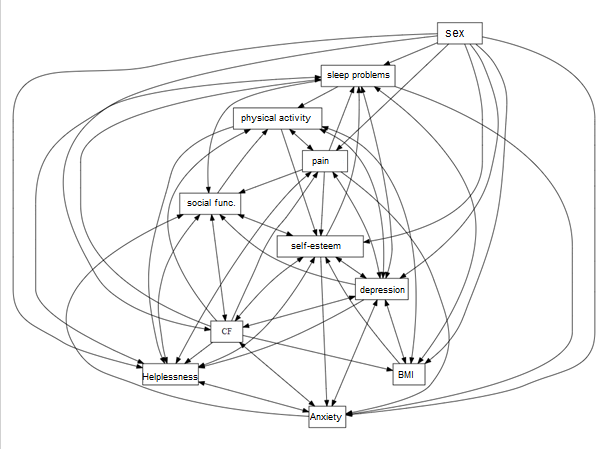
*

*Abbreviations: CF= Chronic fatigue; BMI=Body Mass Index*

**References used in Supplement**

1. Claassen, T. and T. Heskes, *A Bayesian approach to constraint based causal inference*, in *Proceedings of the Twenty-Eighth Conference on Uncertainty in Artificial Intelligence*. 2012, AUAI Press: Catalina Island, CA. p. 207–216.

2. Nogueira, A.R., et al., *Methods and tools for causal discovery and causal inference.* Wiley interdisciplinary reviews: data mining and knowledge discovery, 2022. **12**(2): p. e1449.

3. Bucur, I.G., *Being Bayesian About Causal Inference*. 2020, Radboud University Nijmegen: Nijmegen.

4. Kuipers, J., G. Moffa, and D. Heckerman, *Addendum on the scoring of Gaussian directed acyclic graphical models.* The Annals of Statistics, 2014. **42**(4): p. 1689-1691, 3.

5. Bower, J.E., et al., *Fatigue in long-term breast carcinoma survivors: a longitudinal investigation.* Cancer, 2006. **106**(4): p. 751-8.

6. Nijrolder, I., S.S. Leone, and H.E. van der Horst, *Explaining fatigue: an examination of patient causal attributions and their (in)congruence with family doctors' initial causal attributions.* Eur J Gen Pract, 2015. **21**(3): p. 164-9.

7. Reinertsen, K.V., et al., *Predictors and course of chronic fatigue in long-term breast cancer survivors.* J Cancer Surviv, 2010. **4**(4): p. 405-14.

8. Servaes, P., et al., *The course of severe fatigue in disease-free breast cancer patients: a longitudinal study.* Psychooncology, 2007. **16**(9): p. 787-95.

9. Wareham, N.J., et al., *Validity and repeatability of a simple index derived from the short physical activity questionnaire used in the European Prospective Investigation into Cancer and Nutrition (EPIC) study.* Public Health Nutr, 2003. **6**(4): p. 407-13.

10. AINSWORTH, B.E., et al., *Compendium of Physical Activities: classification of energy costs of human physical activities.* Medicine & Science in Sports & Exercise, 1993. **25**(1): p. 71-80.

11. Bruil, J., Fekkes, M., Vogel, T., Verrips, GHW. *TAAQOL manual*. 2004; Available from: <https://www.tno.nl/media/4727/vragenlijsten_01032012.pdf>.

12. Buysse, D.J., et al., *The Pittsburgh Sleep Quality Index: a new instrument for psychiatric practice and research.* Psychiatry Res, 1989. **28**(2): p. 193-213.

13. Beck, S.L., et al., *Psychometric evaluation of the Pittsburgh Sleep Quality Index in cancer patients.* J Pain Symptom Manage, 2004. **27**(2): p. 140-8.
